# Factors Determining Improvement in Stereopsis and Binocularity After Good Postoperative Alignment in Patients With Childhood-Onset Strabismus

**DOI:** 10.7759/cureus.21964

**Published:** 2022-02-06

**Authors:** Anupam Singh, Nisheeta Patnaik, Sanjeev K Mittal, Ajeet S Bhadoria, Rakesh Panyala, Ramanuj Samanta, Barun Kumar, Omna Chawla

**Affiliations:** 1 Ophthalmology, All India Institute of Medical Sciences, Rishikesh, Rishikesh, IND; 2 Community and Family Medicine, All India Institute of Medical Sciences, Rishikesh, Rishikesh, IND; 3 Cardiology, All India Institute of Medical Sciences, Rishikesh, IND; 4 Physiology, Government Doon Medical College, Dehradun, IND

**Keywords:** binocularity, postoperative alignment, strabismus, childhood onset, stereopsis

## Abstract

Introduction: The purpose of the present study was to determine the factors that affect the outcome of strabismus surgery in terms of improvement in stereopsis and binocularity.

Methods: Data were collected prospectively from patients with childhood-onset, concomitant, constant strabismus greater than 30 prism diopters (PD) with postoperative alignment within 10 PD. Pre- and postoperative stereopsis and binocularity testing were performed using the Titmus fly test, random dot test, Bagolini striated glass test, and Worth four dot test at one, four, and 12 weeks postoperatively.

Results: A total of 73 patients (55% males and 45% females) who underwent surgery at our center were studied. The mean age at the time of surgery was 16 ± 7.7 years (range: 5-35 years). We found that factors such as age of strabismus onset, type of deviation, and amblyopia had a statistically significant influence on the postoperative surgical outcome. A statistically insignificant relationship was noted with gender, refractive error, and duration of strabismus. Patients who had strabismus after six months of age noticed a significant improvement in stereopsis (p-value = 0.000) than those who had strabismus before six months of age (p*-*value = 0.660). Further, there was a statistically significant improvement in patients having exotropia (p-value = 0.018) or combined horizontal and vertical deviations (p*-*value = 0.000), but there was no significant improvement in patients with esotropia (p*-*value = 0.180). Moreover, non-amblyopes had a significantly better postoperative stereopsis than amblyopes (p-value = 0.006). Although there was no association between preoperative deviation and improvement in stereopsis (p-value = 0.081), patients having preoperative deviation in the range of 31-45 PD had a statistically significant improvement in stereopsis (p-value = 0.000). There was no significant difference between postoperative residual deviation and final stereopsis (p-value > 0.05). All the results were the same for both the Titmus test and the random dot test. Binocular fusion was observed in 34 subjects, and uniocular suppression was noted in 38 subjects preoperatively. It was observed that only one patient gained binocular single vision postoperatively.

Conclusion: The presence of amblyopia, esotropia, early onset of strabismus (within six months of age), and a larger preoperative deviation (>45 PD) were associated with poorer stereopsis. In patients with horizontal strabismus, the coexistence of vertical deviation had a positive impact on the postoperative stereopsis. Gender, refractive error, and duration of strabismus did not influence the final stereopsis in our study.

## Introduction

Under normal day-to-day visual conditions, stereopsis and binocular vision are essential for a unique perception of depth. This depends on adequate vision in both eyes, proper oculomotor control, and preserved sensory cortical fusion. The information of depth perception is based on the disparity caused by the images produced in each eye and reaches the primary visual cortex for comparison [1]. This binocular disparity is translated by the visual system as depth perception; however, patients with strabismus have a defective in-depth perception, and their binocular summation is also affected [2].

Infants of around three months of age have cyclopean stereovision, which develops in binocular vision by the age of 10 years [3]. This long period of growth implies that disruption in stereovision can occur during this period; however, it should be realized that any surgical or nonsurgical interventions in these patients during this time give a good recovery window. Early alignment of both the visual axis irrespective of the type of intervention is important for recovery [4] and better outcomes in terms of stereopsis [5-10]. Cosmetic correction of strabismus is always desirable, but binocular recovery and stereopsis are also important, as they add the three-dimensional aspect to vision that not only expands the visual field but also gives a depth perception essential for a child’s reading and writing skills [11]. Moreover, proper and symmetrical ocular movements improve binocular vergences required for accommodation [12]. Early intervention can hasten the path to regaining all these dimensions of vision and thus improve the quality of life of the patient [13]. Few studies on surgical outcomes of strabismus have shown that postoperative alignment, duration of strabismus prior to surgery, presence of amblyopia, type of deviation, and amount of preoperative deviation influence the stereopsis and binocular outcome of the patient [14-17]. Despite this, there is a lack of clarity about the factors that influence postoperative stereoacuity in spite of good alignment after surgery.

Therefore, we assessed the improvement in stereopsis and binocularity after strabismus surgery in patients with a constant deviation of more than 30 prism diopters (PD) with a history of onset of strabismus before three years of age. We examined the patients’ level of stereopsis and binocularity pre- and postoperatively and report the factors that affected the same.

## Materials and methods

The study adhered to the Declaration of Helsinki. After approval by the Institutional Ethics Committee, this prospective observational study was conducted from January 2019 to February 2020 at a tertiary healthcare facility in Northern India.

It was a prospective observational study that included 73 consecutive patients with childhood-onset (before <3 years of age), concomitant constant strabismus (>30 PD) who had successful postoperative alignment after taking informed consent. Successful alignment was defined as primary position alignment within 10 PD at distance and near fixation, as measured by the simultaneous prism and cover test at three months after surgery. Patients were excluded if they had previous ocular disease or surgery, a history of ocular trauma, and a developmental or neurologic abnormality. The informed consent form to participate in the study was signed by patients or patients’ parents or guardians.

Preoperative patient characteristics included gender, age, duration of strabismus, deviation at a distance of 6 m and 33 cm, fixation preference, refractive errors, presence of amblyopia, A or V pattern, dissociated vertical deviation, vertical deviation, and oblique muscle overaction. The presence of fixation dominance was determined with repeated examinations of the cover-uncover test. Refractive error was determined using cycloplegic refraction with atropine 1% or cyclopentolate hydrochloride 1% and analyzed as spherical equivalent values.

All patients underwent a comprehensive ophthalmic examination, including visual acuity assessment using the Snellen’s or Tumbling E or LED visual acuity chart, a complete slit-lamp examination to rule out any anterior segment pathologies, and dilated fundus examination to note posterior segment pathologies and torsional components. The preoperative deviation was measured using the alternate prism cover test, performed at 6 m or beyond for distant fixation and 30 cm for near fixation in primary gaze. Post-occlusion prism bar cover test (PBCT) after one hour of monocular occlusion and another post-occlusion near measurement were obtained with an additional +3.00 D sphere over each eye in patients with good binocular fusion. Preoperative nine gaze photographs of the patient were taken for records.

All preoperative measurements (with appropriate refractive correction) of ocular deviation (using the prism alternate cover or modified Krimsky test), postoperative measurements of ocular deviation, and strabismus surgeries were performed by a single strabismus surgeon. Preoperative stereopsis and binocular single vision testing were performed using the random dot, Titmus stereotest, distant Worth four dot test, and Bagolini striated glass test by a single ophthalmologist after neutralizing the deviation with appropriate prisms. All these tests were repeated without prism neutralization at one, four, and 12 weeks postoperatively.

Stereopsis was first examined with a set of polaroid glasses with the Titmus test, followed by the random dot test. Threshold stereoacuity was recorded in seconds of arc. When the patient consistently reported a level incorrectly, the last correct circle was considered as their threshold stereoacuity. The results were record­ed as “nil” when patients could not identify the pic­ture of 800 seconds of arc. It was then given the arbitrary number of 3000 and 1000 for Titmus and random dot tests, respectively.

Bagolini striated glasses were then provided, and the patient was asked to look at a small light source. If the patient reported two crossed streaks, the test was considered to be positive and said to have fusion. If not, the patient was said to have suppression. Distant Worth four dot test was conducted with a pair of red-green glasses at 6 m. If the patient reports four dots (two green, one red, and one white), the patient was said to have fusion. If the patient sees either three green or two red, they were said to have suppression.

Descriptive analysis using percentage and frequencies were performed in all data. First, for checking the normality of the data set, the Shapiro-Wilk test was applied. All variables were found to be non-normal. For continuous variables, median and interquartile range (IQR) were calculated. Differential analyses were conducted using the nonparametric Mann-Whitney U test and the Kruskal-Wallis test. Statistical assessments were performed using the Statistical Package for the Social Sciences (SPSS) version 22.0 (IBM Corporation, Armonk, NY, USA). The test of significance was two-sided and was set at 5% (p-value ≤ 0.05). The sample size (N = 73) was calculated based on a = 0.05 and b = 0.2. Therefore, the power of the study was 90.

## Results

Among the 73 patients, 55% were males and 45% were females. The mean age at the time of surgery was 16 ± 7.7 years (range: 5-35 years). The patients were divided into three age groups: 0-5 years, 5-10 years, and >10 years; the number of patients in these groups was two, 16, and 55, respectively. All 73 patients achieved postoperative alignment of <10 PD, wherein 14 of them had within 4 PD. The baseline characteristics of the study patients are summarized in Table 1.

**Table 1:** Baseline Characteristics of the Study Population

Parameter	Category	N	Percentage (%)
Gender	Male	40	54.79
	Female	33	45.21
Age	<5 years	2	2.70
	5–10 years	16	21.90
	>10 years	55	75.30
Type of deviation (three criteria)	Esotropia	6	8.22
	Exotropia	22	30.14
	Combined deviation	45	61.64
Type of deviation (four criteria)	Esotropia	6	8.22
	Exotropia	22	30.14
	Esotropia+V	16	21.92
	Exotropia+V	29	39.73
Amblyopia	Present	36	49.32
	Not present	37	50.68
Age of onset of strabismus	<6 months	26	35.62
	6 months–3 years	47	64.38
Preoperative deviation	31–45 PD	43	58.9
	46–60 PD	16	21.92
	61–75 PD	11	15.07
	76–90 PD	3	4.11
Postoperative deviation	<4 PD	14	19.18
	4–10 PD	59	80.82

There was a statistically significant improvement in stereopsis following strabismus surgery at 12 weeks of follow-up in both male and female patients (p-value < 0.05). There was no significant relationship between the baseline stereopsis, final stereopsis, and gender (p-value = 0.544 and 0.326, respectively). Patients aged 5-10 years and >10 years had a statistically significant improvement in stereopsis after strabismus surgery than those aged <5 years (p-value = 0.008, 0.000, and 0.180, respectively). Further, there was a statistically significant improvement in patients having exotropia (p-value = 0.018) or combined horizontal and vertical deviations (p-value = 0.000), but there was no significant improvement in patients with esotropia (p-value = 0.180). There were 36 amblyopes in the study group, and all of them had a statistically significant improvement in median stereopsis (p-value = 0.018), but non-amblyopes had a significantly better improvement than amblyopes (p-value = 0.006) (Table 2).

**Table 2:** Significant Associations of Final Stereopsis On Titmus Test ^a^p-value is obtained by applying the Wilcoxon signed-ranked test (preoperative and postoperative) for each category of the parameter ^b^p-value is obtained by applying the Mann–Whitney U Test/Kruskal–Wallis test for comparing the group median for both, preoperative and postoperative scores for each categorical variable *p-value < 0.05 (significant at 5% level of significance) **p-value < 0.01 (significant at 1% level of significance)

Parameter	Category	Median	IQR		Median	IQR	
Gender	Male	800	400–2450		400	200–2450	0.003**
	Female	400	200–3000		400	100–3000	0.000**
	p-value^b^	0.544			0.326		
Age	<5 years	600	300–600		250	75–300	0.180
	5–10 years	400	155–800		200	85–800	0.008**
	>10 years	800	400–3000		800	200–3000	0.000**
	p-value^b^	0.184			0.036*		
Type of deviation (3 criteria)	Esotropia	800	700–3000		800	400–1350	0.180
	Esotropia	800	140–3000		800	100–3000	0.018*
	Combined	400	400–1900		400	200–1900	0.000**
	p-value^b^	0.415			0.539		
Type of deviation (4 criteria)	Esotropia	800	700–3000		800	400–1350	0.180
	Esotropia	800	140–3000		800	100–3000	0.018*
	Esotropia + V	800	400–800		400	200–800	0.016*
	Esotropia + V	400	400–3000		400	170–3000	0.003**
	p-value^b^	0.556			0.727		
Amblyopia	Present	800	400–3000		800	400–3000	0.018
	Not present	400	300–800		200	120–800	0.000**
	p-value^b^	0.023*			0.006**		
Age of onset of strabismus	<6 months	800	400–3000		800	400–3000	0.660
	6 months–3 years	400	400–800	400		140–800	0.000**
	p-value^b^	0.145			0.029*		
Preoperative deviation	31–45 PD	400	200–800		400	100–800	0.000**
	46–60 PD	800	500–3000		800	400–3000	0.066
	61–75 PD	400	400–3000		400	400–3000	0.157
	76–90 PD	3000	400–3000		3000	400–3000	1.000
	p-value^b^	0.189			0.081		
Postoperative deviation	<4 PD	400	140–400		400	80–400	0.102
	4–10 PD	800	400–3000		400	200–3000	0.000**
	p-value^b^	0.024*			0.071		

There was a statistically significant association between the age of onset of strabismus and improvement in stereopsis. Patients who had strabismus after six months of age noticed significantly better stereopsis improvement (p-value = 0.000) than those who had strabismus before six months of age (p-value = 0.660).

There was no significant association between preoperative deviation and final stereopsis (p-value = 0.081), but patients having preoperative deviation in the range of 31-45 PD had a statistically significant improvement in stereopsis (p-value = 0.000), but patients having a deviation of more than 45 PD did not (p-value > 0.05). There were 14 patients in the study population who achieved postoperative horizontal alignment within 4 PD, and the rest of the 59 patients achieved postoperative horizontal alignment within 10 PD. None of the patients had residual vertical misalignment postoperatively. The first group did not achieve any statistically significant improvement in final stereopsis (p-value > 0.05), but those with horizontal alignment within 10 PD had a statistically significant improvement in stereopsis (p-value = 0.00). However, there was no significant difference between postoperative residual deviation and final stereopsis (p-value > 0.05).

A statistically insignificant relationship was noted with refractive error and duration of strabismus (p-value > 0.05). All the results were the same for both the Titmus test and the Random dot test (Table 2 and Table 3).

**Table 3:** Significant Associations of Final Stereopsis on Random Dot Test ^a^p-value is obtained by applying the Wilcoxon signed-ranked test (preoperative and postoperative) for each category of parameters ^b^p-value is obtained by applying the Mann–Whitney U Test/Kruskal–Wallis Test for comparing group median for both, preoperative and postoperative score for each categorical variable *p-value < 0.05 (significant at 5% level of significance) **p-value < 0.01 (significant at 1% level of significance)

Parameter	Category	Stereopsis: random dot test				
		Pre-test		Post-test		p-value^a^
		Median	IQR	Median	IQR	
Gender	Male	700	400–1000	400	200–1000	0.003**
	Female	400	200–1000	400	100–1000	0.001**
	p-value^b^	0.287		0.169		
Age	<5 years	700	300–750	250	75–300	0.180
	5–10 years	400	155–1000	200	77.5–1000	0.011*
	>10 years	1000	400–1000	400	200–1000	0.000**
	p-value^b^	0.131		0.079		
Type of deviation (three criteria)	Esotropia	1000	850–1000	1000	400–1000	0.317
	Esotropia	1000	140–1000	1000	92.5–1000	0.018*
	Combined	400	400–1000	400	200–1000	0.000**
	p-value^b^	0.182		0.267		
Type of deviation (four criteria)	Esotropia	1000	850–1000	1000	400–1000	0.317
	Esotropia	1000	140–1000	1000	92.5–1000	0.018*
	Esotropia + V	400	400–1000	400	200–1000	0.014*
	Esotropia + V	400	400–1000	400	170–1000	0.003**
	p-value^b^	0.286		0.423		
Amblyopia	Present	1000	400–1000	1000	400–1000	0.027*
	Not present	400	300–1000	200	120–1000	0.000**
	p-value^b^	0.008**		0.002**		
Age of onset of strabismus	<6 months	1000	400–1000	1000	400–1000	0.102
	6 months–3 years	400	400–1000	400	100–1000	0.000**
	p-value^b^	0.228		0.026*		
Preoperative deviation	31–45 PD	400	200–1000	400	100–1000	0.000**
	46–60 PD	1000	550–1000	1000	400–1000	0.066
	61–75 PD	400	400–1000	400	400–1000	0.157
	76–90 PD	1000	400–1000	1000	400–1000	1.000
	p-value^b^	0.114		0.102		
Postoperative deviation	<4 PD	400	140–400	400	70–400	0.102
	4–10 PD	1000	400–1000	400	200–1000	0.000**
	p-value^b^	0.042*		0.115		

Binocular fusion was observed in 34 subjects, and uniocular suppression was noted in 38 subjects preoperatively. It was observed that only one patient gained binocular single vision postoperatively. Both Worth four dot test and Bagolini striated glass test showed similar observations.

## Discussion

Our study showed that 25 out of 73 (34.24%) patients had improvement in stereopsis after 12 weeks of strabismus surgery. Our finding was consistent with the study by Mets et al., who reported that 27 of 72 adults showed improvement by Titmus stereoacuity testing [18], and Fatima et al., in which 10 out of 15 adults with longstanding strabismus gained stereopsis in the TNO test [19]. Dickmann et al. reported that six of 20 patients with childhood strabismus gained stereopsis postoperatively [20]. Further, patients older than five years had better final stereopsis (p-value < 0.05) than those younger than five (p-value < 0.18). This could be because of a better understanding of testing and a better level of cooperation by older patients.

The age of onset of strabismus was an important factor in the development of postoperative stereopsis. We have observed that children who developed strabismus after six months of age had a statistically significant improvement in stereopsis. This finding is consistent with that of a recently published study that concluded that earlier manifestation of ocular misalignment leads to poorer final stereopsis [21]. Strabismus in the first few years of life inhibits the development of binocular sensory neurons in the brain [22]. The period of maturation of stereopsis is considered to be the first decade of life, and any defect in binocularity in this critical period could damage the process of stereopsis maturation. This can be interpreted in terms of the importance of earlier intervention to support neuronal plasticity in the final outcome of good stereopsis.

The presence of amblyopia has been reported to influence the outcome of strabismus study. We also found that the non-amblyopes had a significantly better improvement in stereopsis than amblyopes. It is already known that amblyopia is often associated with the suppression of inputs from the amblyopic eye to the visual cortex. This causes impairment of binocular visual functions at the cortical level. Surgical correction can only provide the conditions necessary for reestablishing full binocular function in strabismus [23].

Moreover, most of the patients in our study pool had strabismic amblyopia, and it has been postulated that stereopsis is more affected in strabismic than in anisometropic amblyopia [24]. This further supports the surgical alignment to achieve better stereopsis. Poor final stereopsis of amblyopic patients may be attributed to the fact that patients with strabismic amblyopia have a remote possibility of improvement with single surgical intervention due to the destructive effect of amblyopia on potential final stereoacuity; they may have required more direct stereo training to achieve better stereopsis rather than monocular training [24].

Those with esotropia had poorer final stereopsis than those with exotropia or combined horizontal and vertical deviation. This finding is partially consistent with that of previously published studies that concluded that exotropia had a better improvement in stereopsis as opposed to esotropes, but there was no statistically significant association between the type of deviation and final stereopsis [15,21]. In our study, a statistically significant improvement was seen in the combined group in contrast to the previously published studies [21]. This could be due to the correction of vertical and torsional components of the deviation; however, it requires further validation by future studies.

Further, it was interesting to observe that preoperative deviation had no significant association with stereopsis improvement. However, on the subgroup analysis, we report that the value of preoperative deviation is significant as patients with preoperative deviation in the range of 31-45 PD had a statistically significant improvement in stereopsis (p-value = 0.000) compared with those having a deviation of more than 45 PD (p-value > 0.05). This finding is partly consistent with a study by Eshaghi et al., who concluded that a higher amount of ocular deviation is associated with immature stereopsis [21].

We found no association between postoperative residual deviation and final stereopsis (p-value > 0.05), in contrast to the previously published studies [15,21]. There were 14 patients in the study population who achieved postoperative horizontal alignment within 4 PD, and the rest of the 59 patients achieved postoperative horizontal alignment within 10 PD. None of the patients had residual vertical misalignment. The first group did not achieve any statistically significant improvement in final stereopsis (p-value > 0.05), but those with horizontal alignment within 10 PD had a statistically significant improvement in stereopsis (p-value = 0.00); this was because of the fact that the group that had horizontal alignment within 4 PD had better baseline stereopsis as compared with the other group (p-value < 0.05) (Table 2 and Table 3). The improvement in stereopsis in individuals with residual deviation within 10 PD needs further validation in future studies.

Our study reports no statistically significant association between the type of refractive error and improvement in postoperative stereopsis. This is in contrast with the report of Kim et al. and Leffler et al., who concluded that hyperopic refractive errors are a good prognostic factor in intermittent exotropia surgeries [25,26].

In our study, a reversal of uniocular suppression was seen only in one patient (1.3%), which is very low as compared with the findings of Kushner et al., where 86% of 359 consecutive adults with strabismus gained postoperative binocularity [6]. Similarly, Scott et al. and Birch et al. showed significantly better improvement in binocularity [12,27]. Conversely, Ganguly et al. reported that the restoration of binocularity occurred only in three of 40 adults [28]. This disparity in the findings across researchers can be due to the differences in the approach for binocular assessment, and the age group is also not comparable; the study of Kushner et al. dealt with adults, and the other two have the assessment of binocularity at 12 months postoperative.

The findings of our study are significant for predicting the outcome of surgical management of strabismus. However, these should be interpreted with some of our limitations: the small size of the study sample and the follow-up period of only 12 weeks. We feel that the subjects with amblyopia could also be further analyzed with the addition of amblyopia therapy, which was beyond the scope of this study. The prospective study design is the main strength of the study with a wide variety of strabismus types, age groups, and refractive errors, leading to sample heterogenicity that can reflect real situations as each patient comes with unique combinations.

## Conclusions

In conclusion, we report that there was a significant improvement in stereopsis following strabismus surgery in 34.24% of the study pool. The presence of amblyopia and esotropia, the onset of strabismus within six months of age, and a larger preoperative deviation (>45 PD) were associated with poorer stereopsis. In patients with horizontal strabismus, the coexistence of vertical deviation had a positive impact on postoperative stereopsis. Gender, refractive error, and strabismus duration do not influence the postoperative achievement of stereopsis. The restoration of the binocularity is tough after strabismus surgery in adults with childhood-onset strabismus. Thus, we recommend that early surgical intervention contributes to better stereopsis and should be encouraged. Further studies with longer follow-up are required to establish these findings with certainty.
